# Effect of decompression-induced bubble formation on highly trained divers microvascular function

**DOI:** 10.1002/phy2.142

**Published:** 2013-11-07

**Authors:** Kate Lambrechts, Jean-Michel Pontier, Aleksandra Mazur, Peter Buzzacott, Jean Morin, Qiong Wang, Michael Theron, Francois Guerrero

**Affiliations:** 1 Orphy Laboratory, Université de Bretagne Occidentale29200, Brest, France; 2 Diving and Hyperbaric Department, French Navy Diving SchoolBP 311, 83800, Toulon, France

**Keywords:** Decompression, endothelium, laser Doppler flowmetry, microcirculation

## Abstract

We previously showed microvascular alteration of both endothelium-dependent and -independent reactivity after a single SCUBA dive. We aimed to study mechanisms involved in this postdive vascular dysfunction. Ten divers each completed three protocols: (1) a SCUBA dive at 400 kPa for 30 min; (2) a 41-min duration of seawater surface head immersed finning exercise to determine the effect of immersion and moderate physical activity; and (3) a simulated 41-min dive breathing 100% oxygen (hyperbaric oxygen [HBO]) at 170 kPa in order to analyze the effect of diving-induced hyperoxia. Bubble grades were monitored with Doppler. Cutaneous microvascular function was assessed by laser Doppler. Endothelium-dependent (acetylcholine, ACh) and -independent (sodium nitroprusside, SNP) reactivity was tested by iontophoresis. Endothelial cell activation was quantified by plasma Von Willebrand factor and nitric oxide (NO). Inactivation of NO by oxidative stress was assessed by plasma nitrotyrosine. Platelet factor 4 (PF4) was assessed in order to determine platelet aggregation. Blood was also analyzed for measurement of platelet count. Cutaneous vascular conductance (CVC) response to ACh delivery was not significantly decreased by the SCUBA protocol (23 ± 9% before vs. 17 ± 7% after; *P* = 0.122), whereas CVC response to SNP stimulation decreased significantly (23 ± 6% before vs. 10 ± 1% after; *P* = 0.039). The HBO and immersion protocols did not affect either endothelial-dependent or -independent function. The immersion protocol induced a significant increase in NO (0.07 ± 0.01 vs. 0.12 ± 0.02 *μ*g/mL; *P* = 0.035). This study highlighted change in microvascular endothelial-independent but not -dependent function in highly trained divers after a single air dive. The results suggest that the effects of decompression on microvascular function may be modified by diving acclimatization.

## Introduction

During decompression sickness (DCS) inert gas (usually nitrogen) supersaturation results in the formation of intravascular bubbles which can lead to venous gas emboli (VGE). Doppler ultrasonic detection of circulating VGE after diving is considered a useful index for safe decompression (Nishi et al. 2003). Generally, it has been assumed that venous bubbles will be trapped in the pulmonary circulation and have no further effects on the arterial circulation. Although in most cases VGE occur without acute clinical symptom of DCS, decompression-induced bubble formation is the pivotal event in DCS. However, recent literature showed implication of different postdive pathophysiological events as endothelium dysfunction, coagulation, and inflammation processes that could play a substantial role in the occurrence of DCS. Circulating bubbles affect the clotting system both through activation of the coagulation cascade and the induction of platelet aggregation (Pontier et al. 2009b). They also trigger intravascular neutrophil activation through elevation in the number of circulating annexin V-coated microparticles derived from leukocytes, erythrocytes, platelets, and endothelial cells (Yang et al. 2012). Bubble-platelet aggregates can occlude blood vessels and cause vascular endothelial cell stripping through mechanical interaction with the endothelium, causing damage (Nossum et al. 1999; Barak and Katz 2005).

Other studies have shown that VGE cause a reduction in endothelial function of the pulmonary artery in pigs and rabbits (Nossum et al. 1999, 2002). Similarly, in healthy divers without any DCS symptoms, a single air dive has been repeatedly reported to reduce the flow-mediated dilation (FMD), a marker of endothelial function, of the brachial artery (Brubakk et al. 2005; Theunissen et al. 2013).

Recently, we reported that endothelial-dependent vasorelaxation is decreased in microvascular circulation after an open sea air dive (Lambrechts et al. 2013), similar to the effect on conductance arteries. However, in the previous study we also found that endothelium-independent vasorelaxation, as assessed by the response to nitric oxide (NO) donors, is decreased in both the brachial artery and microcirculation after a single air dive (Lambrechts et al. 2013). This finding suggests that SCUBA air diving could impair not only the endothelium but also vascular smooth muscle function.

Impairment of microvascular function could play a pivotal role in end-organ dysfunction associated with metabolic and cardiac diseases (Lockhart et al. 2009). Besides its role in vascularization and metabolic exchanges between blood and tissue, microcirculation ensures the regulation of the inflammatory process, blood pressure, and the control of tissue fluid and edema (Gore and Bohlen 1977). All these actions are known to be influenced by the constraints induced during diving (Ersson et al. 1998; Huang et al. 2003; Perymon et al. 2005).

In the brachial artery, impairment of FMD lasted for 72 h after a single air dive and was partially reversed by acute and long-term predive supplementation of antioxidants, implicating oxidative stress as an important contributor to the postdive vascular dysfunction (Obad et al. 2007b). Among several mechanisms which can account for generation of reactive oxygen species (ROS), hyperoxia has been repeatedly cited. Increased formation of ROS can reduce the bioavailability of NO, either by impairing the function of NO synthase (Lakshminrusimha et al. 2007) or by rapidly reacting with it to form peroxynitrite (Rubanyi and Vanhoutte 1986) which in turn leads to the production of nitrotyrosine (NT) (a product of tyrosine nitration). NT is detected in a large number of pathological conditions (Pacher et al. 2005; Mohiuddin et al. 2006) and is considered as a marker of NO-dependent, reactive nitrogen species-induced nitrative stress.

Habituation to stress decompression has been reported to decrease bubble formation (Pontier et al. 2009a) and protect from DCS in humans (Hagberg and Ornhagen 2003; Cameron et al. 2007) as well as in animal models (Su et al. 2004; Arieli et al. 2007). Postulated mechanisms include the depletion of complement proteins, thus preventing a massive activation of the complement system (Nyquist et al. 2007). Others studies propose the accumulation of protective factors such as early nonspecific stress markers HSP27 (Montcalm-Smith et al. 2007) or HSP70 (Su et al. 2004).

In this study, we aimed to highlight the mechanisms involved in this postdive vascular dysfunction. For this we assessed human cutaneous endothelial function in healthy, highly trained divers after an open-sea air dive using laser Doppler flowmetry (LDF). To understand the mechanisms involved in microvascular changes, we measured postdive plasmatic markers of endothelial activation and/or damage, in relation to bubble formation and platelet activation. Because air SCUBA diving implies both immersion and hyperbaric oxygen (HBO), we also assessed the effects upon microvascular function during a single surface head immersed finning exercise or HBO exposure.

## Method

### Study population

Ten military divers (age: 35 ± 7 years; body mass index: 24 ± 1 kg m^−2^) volunteered for this study. The subjects were experienced divers with a mean of 1493 dives and certified medically fit for diving. None had previously experienced DCS. Prior to the experimental protocol, subjects abstained from any physical activity and diving for 48 h. None of the participants in this study used any drugs. Tea, coffee, alcohol, and smoking were prohibited for 6 h prior to the test. All protocols were performed in the morning. Potential risks were explained to each subject in detail and they gave written informed consent before the experiment. The protocol was approved by the Committee on the Ethics of Research in Human Experimentation of Marseille and was performed in accordance with the guidelines of the Helsinki Declaration for human research.

### Study design

The design was a randomized *before and after* study and each subject was his own control. Each diver performed three experimental protocols separated by at least 72 h, in a randomly allocated order.

The “SCUBA” protocol consisted in an open-sea air dive in field conditions (water temperature: 15 ± 1°C). The diving site was located in the relative vicinity of the military diving school, and divers were transported to the site by a power boat during a maximum of 10-min ride. Divers descended to 30-m seawater (msw) depth (400 kPa pressure). After 30-min exposure (mostly at 30-msw depth following descent at ∼150 kPa-min^−1^), with moderate finning exercise, the rate of decompression was 150 kPa-min^−1^ with a 9-min stop at 3 msw according to the French Navy procedure (MN90). Subjects used masks and fins and were dressed in 5-mm neoprene wet suits with hood, boots, and gloves. Depth and dive time were monitored by each diver's personal dive computer. The immersion protocol consisted of a 41-min immersion (temperature 15 ± 1°C) at atmospheric pressure. In this protocol, subjects were surface head immersed, breathing air, and performing a moderate finning exercise. For these in-field protocols, subjects used masks and fins and were dressed in a 5-mm neoprene wet suit with hood, boots, gloves, and a thin neoprene top. To assess the effect of hyperoxia alone, a HBO protocol consisting of a 41-min simulated dive at 170 kPa in a dry chamber breathing 100% oxygen, (i.e., same oxygen pressure as during the SCUBA protocol), followed by decompression at the rate of 100 kPa min^−1^ was carried out.

### Bubbles analysis

Circulating vascular bubbles were monitored in the pericordial area by an experienced investigator both with the divers at rest and after knee flexions. Each measurement lasted for 3 min and was performed with a pulsed Doppler equipped with a 2 MHz probe (Pioneer® Siemens, Malvern, PA) at 30, 60, and 90 min after surfacing. The Spencer scale was used to rate the bubble signal. The Kisman integrated severity score (KISS) was calculated according to the following formula (Eftedal and Brubakk 1997):


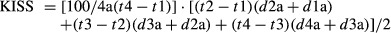


where *t* = time of observation in minutes after reaching surface (30, 60, and 90 min), *d* = doppler score (grades 0–4; 0 = occasional bubble signal, 4 = continuous bubble signal) observed at time *t*, and a = 3 (the parameter a accounts for the nonlinear measure of bubble grade).

### Laser Doppler flowmetry

In order to assess the cutaneous microvascular endothelial function, we performed iontophoresis with pharmacological agents coupled with LDF. In all protocols this measurement was performed for each subject in the supine position in a controlled temperature room (22 ± 1°C) before diving and 30 min after surfacing (Brubakk et al. 2005). Each subject was therefore used as his own control. Iontophoresis allows for local delivery of small amounts of pharmacological agents, thus avoiding potential systemic effects while delivering drugs in the area of blood flow measurement. The cutaneous blood flow response to iontophoresis was assessed in the forearm using a LD probe. We used a specially designed probe (PF 450-PI; Perimed, Järfälla, Sweden) to allow for current application and simultaneous cutaneous blood flow recording. The probe had a chamber where we positioned a 0.6 cm^2^ sponge. At the center of the sponge cutaneous LDF was measured through a multifiber laser probe (780 nm). The iontophoretic sponge was connected to regulated current suppliers (Perilont PF 382; Perimed), allowing for the delivery of regulated-intensity currents for programmable durations.

**Figure 1:**
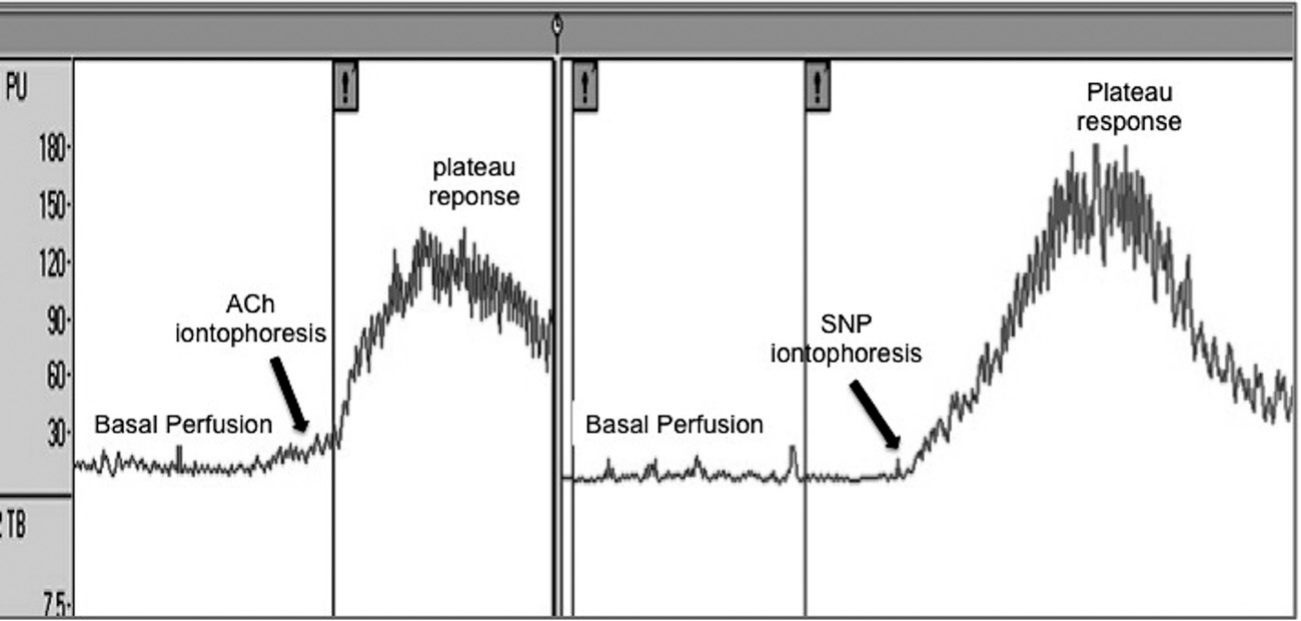
Example of laser Doppler flowmetry recording, showing basal perfusion, increased perfusion after iontophoresis stimulation until reaching plateau response.

Iontophoresis involves the use of a low electrical current to deliver pharmacological agents through the skin. For endothelium-dependent and endothelium-independent vasodilation analysis, we measured blood flow changes firstly in response to 1% ACh chloride solution, (to study endothelium-dependent vasodilation), and then to 1% SNP, (to study endothelium-independent vasodilation) (Heylen et al. 2008). These measurements were performed with the application of an anodal current of 35 sec, 10 mA for the experiment with ACh and a 35 sec, 10 mA cathodal current for the experiment with SNP. A stable baseline blood flow was measured for 2 min before the current was applied. Measurement of the peak increase in LD flux (perfusion) was performed at the peak of the maximal plateau reached after each stimulation, in order to represent the increase in vasodilation (Fig. 1). Cutaneous blood flow was indexed as was cutaneous vascular conductance (CVC), calculated as LD flux/mean arterial pressure (CVC = LD/MAP = mV/mmHg). Mean arterial blood pressure (MAP) was measured using a sphygmomanometer and stethoscope, and was taken to be the mean value obtained from two blood pressure measurements recorded at rest just before each LD assessment (MAP = [(2 × diastolic) + systolic]/3). Responses to ACh and SNP were presented as percentage of CVC variation between baseline and iontophoretic response.

The LDF signal intensity depends on velocity and concentration of moving blood cells in the site under examination. On the other hand, plasma volume changes have been previously reported after SCUBA diving (Coulange et al. 2008; Jimenez et al. 2010). Therefore, in order to exclude any error of measurement due to decrease in plasmatic volume, in additional experiments we assessed the effect of hemoconcentration on the response of skin perfusion elicited by iontophoresis of either ACh or SNP. Skin blood flow was measured in nine healthy subjects before and 60 min after administration of 40 mg of Lasilix, a diuretic drug. In order to assess any variation in plasmatic volume, blood was analyzed in duplicate for measurements of red blood cell, hematocrit, and platelet count (PC) with a blood cell counter (I-stat System; Abbott Point of Care Inc., Princeton, NJ).

### Blood sampling technique and measurements

Measurements of plasmatic values for nitrites, NT, Von Willebrand factor (VWf), and platelet factor 4 (PF4) were performed in the three protocols for each subject. All blood samples were obtained in the morning (08.00 am–12.00 pm) eliminating possible diurnal variations. Blood samples were drawn 1 h before and 1 h after the end of exposure (SCUBA dive, HBO, or immersion) from a clean median antecubital venepuncture using a 21 gauge needle and a 4.5 mL Vacutainer® tubes (Becton Dickinson, Franklin Lakes, NJ) with citrate-theophylline, adenosine, dipyridamole for PF4 and with ethylenediaminetetraacetic acid 7.5% anticoagulation for VWf, nitrites and NT analysis.

For determination of plasma nitrite concentrations (*μ*g/mL), blood samples were centrifuged at 3000*g* and 4°C for 10 min, aliquoted, and stored at −80°C until the assay. The method performed was based on a colorimetric reaction, (developed by nitrites dissolved in an acid solution containing sulfanilamide) using the Griess reagent (Giustarini et al. 2008).

Plasmatic level of NT was assessed by measuring 3-nitrotyrosine levels (nmol/L) using the OxiSelect Nitrotyrosine ELISA kit (enzyme-linked immunosorbent assay; Cell Biolabs Inc, San Diego, CA) according to provider instructions.

Analysis for VWf and values of plasmatic level (mU/mL) were determined using the Von Willebrand Factor Human ELISA kit (Abcam, Cambridge, U.K.) according to provider instructions. The PF4 human ELISA kit (Abcam, Cambridge, U.K.) was used to determine PF4 plasmatic concentration (pg/mL). Blood was also analyzed for measurement of PC with a blood cell counter (Sysmex Xe 2100 w; Sysmex Corp., Kobe, Japan).

### Statistical analysis

All data are presented as mean ± SEM. For microvascular endothelial function analyse, responses to ACh and SNP are presented as percentage of variation perfusion after iontophoretic stimulation. For statistical analysis of the tested parameters before and after the dive, we used the Statistica 10 software program (Tulsa, OK). Paired *t*-test was used to compare groups of paired data after Kolmogorov–Smirnov tests for normality. Where data were not normally distributed (PC) Wilcoxon signed rank sum tests were performed. Pearson's correlation was used to investigate linear relationships between variables. Statistical significance was accepted at *P* < 0.05.

## Results

**Figure 2:**
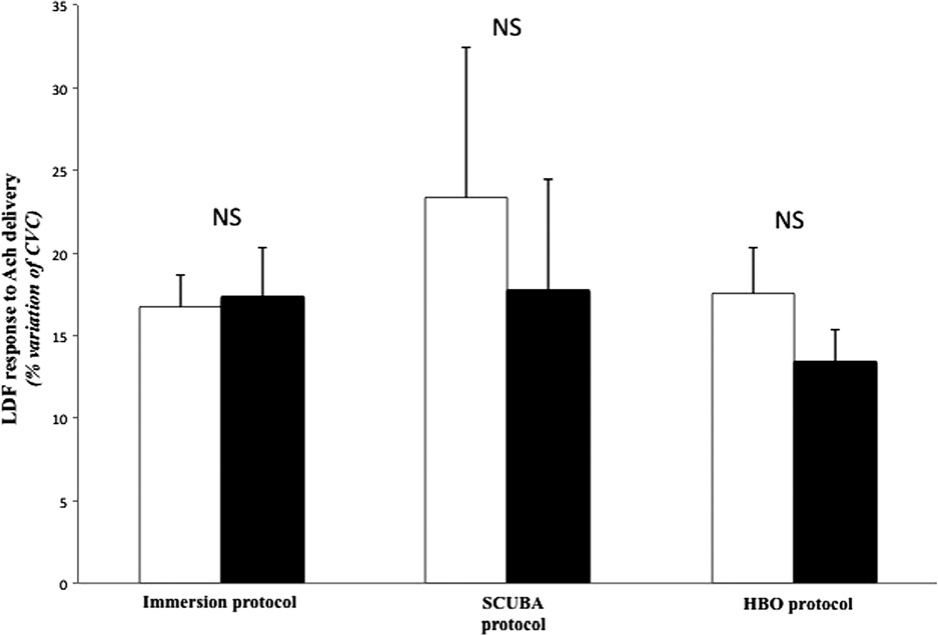
Laser Doppler Flowmetry expressed as a percentage (mean ± SEM) of CVC response to acetylcholine (ACh) iontophoresis stimulation, before (white bars) and after (black bars) each of three protocols (*n* = 10).

**Figure 3:**
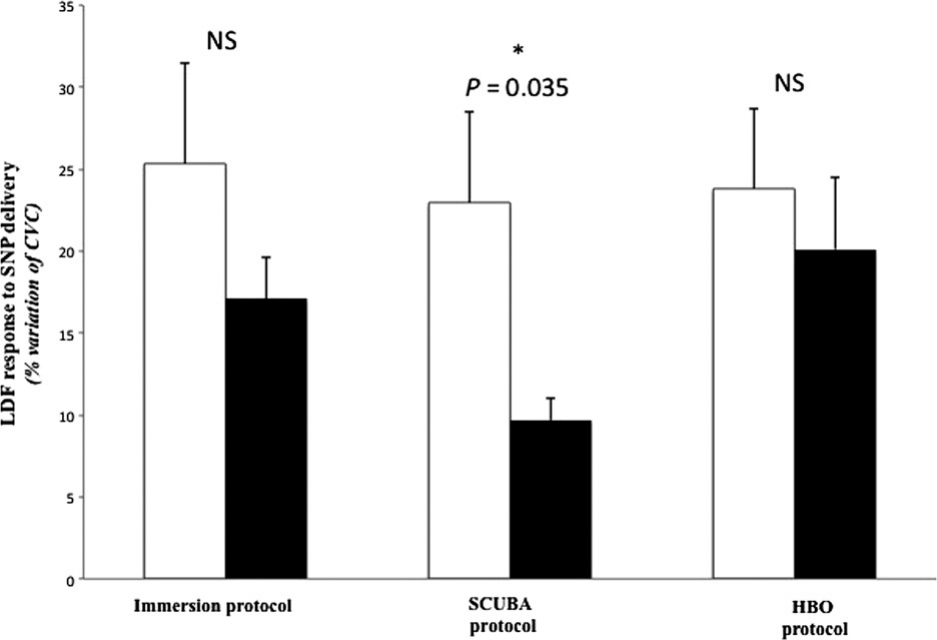
Laser Doppler flowmetry expressed as a percentage (mean ± SEM) of CVC response to sodium nitroprusside (SNP) iontophoresis stimulation, before (white bars) and after (black bars) each of three protocols. *P < 0.05 between before and after the SCUBA protocol (*n* = 10).

Laser Doppler flowmetry results are reported in Figures 2 and 3. Mean KISS bubble score are described in Table 1. For blood samples and markers analysis the mean values and SEM are presented in Table 2 by each protocol.

**Table 1:** Spencer scale and KISS score at rest and after knee flexion (30, 60, and 90 min after the SCUBA protocol)

Subjects	At rest				After knee flexion			
	
	Spencer scale			KISS score	Spencer scale			KISS score
			
	30′	60′	90′		30′	60′	90′	
1	2	1	1	**4.29**	3	3	3	**42.12**
2	0	0	0	**0**	1	1	0	**1.17**
3	0	0	0	**0**	0	0	0	**0**
4	2	2	1	**9.75**	2	3	2	**27.3**
5	2	2	1	**9.75**	4	3	2	**49.14**
6	1	2	1	**7.02**	2	3	2	**27.3**
7	1	1	1	**1.56**	3	3	3	**42.12**
8	0	1	0	**0.78**	1	1	1	**1.56**
9	0	1	1	**1.17**	1	2	2	**9.75**
10	2	1	0	**3.9**	3	2	0	**16.77**
Mean ± SEM	**1 ± 0.29**	**1.1 ± 0.23**	**0.6 ± 0.16**	**3.82 ± 1**	**2 ± 0.39**	**2.1 ± 0.34**	**1.5 ± 0.37**	**21.72 ± 6**

Mean Spencer scales and KISS scores calculated from Spencer scales are presented in bold. Underlined values represent mean kiss scores at rest and after knee flexion.

**Table 2:** Blood markers analysis

	Immersion protocol		HBO protocol		SCUBA protocol	
			
	Before	After	Before	After	Before	After
NO (*μ*g/mL)	**0.07 ± 0.01**	0.12 ± 0.02*	0.116 ± 0.02	0.094 ± 0.02	0.115 ± 0.014	0.155 ± 0.02
NT (n[mol/L]/L)	2.75 ± 0.79	3.57 ± 1.47	2.98 ± 0.81	3.16 ± 1.11	4.27 ± 2.01	2.83 ± 0.87
VWf (mU/mL)	1156 ± 192	1072 ± 132	1211 ± 92	1118 ± 189	766 ± 144	668 ± 112
PF4 (pg/mL)	2862 ± 330	3595 ± 441	3102 ± 380	2932 ± 366	2614 ± 403	2971 ± 388
PC (platelets/*μ*L)	229,207 ± 19,358	226,792 ± 19,790	182,534 ± 28,030	179,523 ± 26,659	221,700 ± 18,080	213,630 ± 16,705

VWf, NT, NO, and PF4 concentrations before and after each protocol. Values are expressed as mean ± SEM (*n* = 10).

* *P* < 0.05 compared with basal value.

Bold value refers to *P* = 0.035.

### SCUBA diving

None of the divers showed any symptoms of DCS in the SCUBA protocol. For bubble score, the mean KISS score was 21.7 ± 6 after knee flexion (Table 1). We measured a reduction of 5.03 ± 0.01% in plasma volume after the dive. Although PC was nonsignificantly decreased (221,700 ± 18,080/*μ*L vs. 213,630 ± 16,705/*μ*L before and after the dive, respectively; *P* = 0.065), we found a significant positive correlation between the KISS bubble score and the decrease in PC after the SCUBA dive (*P* = 0.017). Plasmatic concentration of PF4 was not significantly modified by the SCUBA dive (2615 ± 403 pg/mL vs. 2971 ± 388 pg/mL; *P* = 0.404). There was no difference in conductance baseline values before (0.062 ± 0.007 mV/mmHg) and after (0.057 ± 0.006 mV/mmHg) the dive (*P* = 0.549). Mean CVC response to ACh delivery was not significantly decreased by the SCUBA dive protocol, (from 23 ± 9% predive to 17 ± 7% postdive; *P* = 0.122) (Fig. 2), whereas CVC response to SNP stimulation decreased significantly from 23 ± 6% to 10 ± 1% (*P* = 0.039) (Fig. 3). However, the KISS bubble score did not correlate either to the decrease in response to ACh after the SCUBA dive (*P* = 0.494) or to the variation in response to SNP (*P* = 0.199).

Our results did not highlight any endothelial cell activation as the mean plasmatic VWf concentrations were not significantly changed after the SCUBA protocol (766 ± 144 mU/mL vs. 668 ± 112 mU/mL; *P* = 0.339).

We also found no difference for plasmatic level nitrites (0.115 ± 0.014 *μ*g/mL vs. 0.155 ± 0.02 *μ*g/mL, before and after the dive, respectively; *P* = 0.115) as well as tyrosine nitration (4.27 ± 2.01 n(mol/L)/L vs. 2.83 ± 0.87 n(mol/L)/L; *P* = 0.153).

### Hyperbaric oxygen

For each subject, no circulating bubbles were detected at 30, 60, and 90 min after surfacing. After the HBO protocol PC was nonsignificantly decreased (182,534 ± 28,030 /*μ*L vs. 179,523 ± 26,659 /*μ*L before and after the dive respectively; *P* = 0.621) and PF4 plasma concentration was not modified (3102 ± 380 pg/mL vs. 2932 ± 366 pg/mL; *P* = 0.745). There was no difference in conductance baseline values before (0.064 ± 0.008 mV/mmHg) and after (0.064 ± 0.007 mV/mmHg) the HBO protocol (*P* = 0.945). The CVC responses to ACh (17 ± 2% vs. 13 ± 2%; *P* = 0.247) and to SNP (24 ± 5% vs. 20 ± 4%; *P* = 0.279) were not impaired by oxygen breathing. Plasmatic VWf concentration was not significantly changed (1211 ± 92 mU/mL vs. 1118 ± 189 mU/mL; *P* = 0.589). Plasmatic concentration of nitrite also showed no change after the HBO protocol (0.116 ±0.02 *μ*g/mL vs. 0.094 ± 0.018 *μ*g/mL; *P* = 0.227), as did NT (2.98 ± 0.81 n(mol/L)/L vs. 3.16 ± 1.11 n(mol/L)/L; *P* = 0.481).

### Immersion

PC was not significantly changed (229,207 ± 19,358/*μ*L vs. 226,792 ± 19,790/*μ*L; *P* = 0.625) and PF4 plasma concentration was not modified after immersion (2862 ± 330 pg/mL vs. 3596 ± 441 pg/mL; *P* = 0.441). There was no difference in conductance baseline values before (0.061 ± 0.004 mV/mmHg) and after (0.089 ± 0.007 mV/mmHg) the immersion (*P* = 0.111). The CVC responses to ACh (16 ± 2% vs. 17 ± 3%; *P* = 0.882) and to SNP (25 ± 6% vs. 17 ± 2%; *P* = 0.191) were not impaired by the immersion protocol. There was no significant change in mean plasmatic VWf concentrations (1156 ± 192 mU/mL vs. 1072 ± 132 mU/mL *P* = 0.603). The results showed a significant increase in mean value of plasmatic nitrites levels for the immersion protocol (0.07 ± 0.01 *μ*g/mL vs. 0.12 ± 0.02 *μ*g/mL; *P* = 0.035) but no tyrosine nitration was detected by NT following immersion (2.75 ± 0.79 n(mol/L)/L vs. 3.57 ± 1.47 n(mol/L)/L; *P* = 0.229).

### Dehydration

Despite a reduction of 6.19 ± 0.79% in plasma volume after Lasilix administration, conductance basal values were unchanged (0.07 ± 0.005 mV/mmHg vs. 0.08 ±0.01 mV/mmHg; *P* = 0.108). The CVC response to ACh (27 ± 5% vs. 21 ± 3%; *P* = 0.924) and to SNP (26 ± 8% vs. 26 ± 6%; *n* = 9, *P* = 0.481) remained similarly unchanged.

## Discussion

The first aim was to study human microvascular endothelial function in peripheral circulation of highly trained healthy divers after an open-sea air dive using LDF and iontophoresis, and to relate these modifications to bubble-induced platelet aggregation, endothelial activation/damage, and oxidative stress using the measurements of specific plasmatic markers. Microcirculation is sensitive to a large variety of physiologic stimulants and environmental conditions which are associated with SCUBA diving activities, (namely, immersion, water temperature, physical exercise, hyperbaric hyperoxia, and bubble formation). In the study design, each subject performed three separate protocols at least 72 h apart. In the SCUBA protocol, the subjects performed an air dive in field conditions to evaluate the effect of diving on endothelium-dependent and -independent microvascular function. The HBO protocol was achieved in order to analyze the effect of exposure to hyperbaric oxygen as was induced by the diving conditions. The immersion protocol was used to determine the effect of cold seawater immersion and moderate physical activity.

Our results showed no significant difference in microvascular endothelial function after each protocol, while endothelial-independent function decreased significantly after the SCUBA protocol. Endothelial-independent function remained unchanged after the HBO and immersion protocols. No changes were observed in plasma VWf, PF4, PC, NT, or NO concentrations, except that the immersion protocol induced a significant increase in NO.

The main result highlighted no change in microvascular endothelial function after SCUBA dive, conversely to what we previously reported in recreational divers after the same SCUBA dive protocol (Lambrechts et al. 2013). Coherent with the lack of alteration of the response to iontophoretic ACh, in this study VWf levels did not differ between before and after exposure, showing no endothelial cell activation after SCUBA diving. This was further confirmed by the unchanged plasmatic nitrite and NT levels, indicating that in these subjects NO production was not altered by our diving protocol nor did we detect inactivation by oxidative stress.

In this study, the mean KISS bubble score was lower than found among recreational divers, where KISS bubble scores was 55 after knee flexion (Lambrechts et al. 2013). One might argue that the low magnitude of bubble formation detected in this study may preserve vasculature from endothelial dysfunction or damage. Our subjects were military divers in the French Navy Diving School. All of them were experienced divers with an intensive diving training program during the year including repeated open-sea dives (on average a total of 268 dives per year), as well as intensive physical exercise activity (daily 60-min jogging in field conditions with physical exercise intensity at 50–60% of maximal oxygen uptake, corresponding to aerobic exercise). Some authors have shown that a single bout of strenuous physical exercise before the dive decreased Doppler-detectable gas bubbles after decompression in man (Blatteau et al. 2005). This effect was related on rats to acute effect of physical exercise that was most notable 20 h after a single exercise bout, since it lasted for 48 h (Wisloff and Brubakk 2001). Moreover, this same study has shown that a 6 weeks daily physical training also prevented DCS. Because in our study, subjects were asked to not practice any physical activity for at least 48 h before each protocol, it is unlikely that the lower bubble formation after the SCUBA dive was due to recent physical exercise. Rather, because of their high frequency of diving, the divers in this study could be subject to the so-called diving acclimatization. Diving acclimatization refers to a phenomenon that occurs when individuals undergoing repeated compression–decompression cycles are able to reduce the magnitude of bubble formation (Huang et al. 2003). Indeed, we previously showed that a 3-month intensive training for diving decreased bubble formation after a standard dive (Pontier et al. 2009a) in same military dive trainees population used in this study. Postdive impairment of FMD is probably independent from bubble formation as suggested when decreased FMD even after a single air dive producing few vascular bubbles (Brubakk et al. 2005) as also occurs after breath-hold dives (Theunissen et al. 2013). Thus, the lack of postdive impairment of response to ACh can be due to other mechanisms related to acclimatization. Postulated mechanisms of acclimatization include the depletion of complement proteins, thus preventing a massive activation of the complement system. In humans subjected to different profiles of simulated dives, Nyquist et al. (2007) reported that the decrease in plasmatic concentration of [C3a] correlated with the estimated DCS risk, although the correlation coefficients were low. Rabbits that were observed to have symptoms were also found to have native complement systems that were activated by air bubbles (Ward et al. 1990). In these animals pharmacological complement deactivation prevented the occurrence of DCS symptoms when they were exposed to the same pressure profile. Others studies have proposed the accumulation of protective factors such as early nonspecific stress markers HSP27 (Montcalm-Smith et al. 2007) or HSP70 (Su et al. 2004). Further studies are needed to assess the presence of such mechanisms among trained divers.

Another interesting result was the significantly decreased response to SNP after the SCUBA protocol, confirming previous data from our group (Lambrechts et al. 2013). SNP is generally thought to be an endothelium-independent relaxant agent and its action has been attributed solely to its direct effect on the smooth muscle (Bonaventura et al. 2008). Therefore, one possibility to explain the reduced response to SNP after diving could be an abnormality in vascular smooth muscle. Explanations include a decrease in vascular smooth muscle sensitivity to NO and/or a dysfunction in the relaxation mechanism itself.

Regarding bubble-induced platelet aggregation during decompression, our results confirm those of a previous study (Pontier et al. 2008) with a positive correlation between bubble formation intensity and magnitude of postdive decrease in PC. Today, the evidence for an interaction between platelet and bubbles appears firmly established. Despite this relationship between bubble formation and PC, we did not detect significant platelet aggregation through PF4 assessment after diving. PF4 is a chemokine released from alpha granules of activated platelets during platelet aggregation. In a rat model of decompression PF4 has been used as a marker of platelet aggregation which increased after a provocative dive causing DCS to 59% of the rats (Pontier et al. 2009b). In this study, PF4 did not appear to be a relevant marker of platelet aggregation in humans after safe decompression without DCS. Another explanation could be that the amount of bubbles produced was too low to provoke significant bubble-induced platelet aggregation.

Platelet–vessel wall interactions can occur following endothelial injury (Rumbaut and Thiagarajan 2010). Platelet activation and endothelial function had not previously been tested simultaneously after diving. In this study, PF4 and ACh response did not appear to be modified, excluding, in this case, any deleterious interaction between platelet and endothelial wall.

The HBO protocol did not provoke modification in microvascular function due to hyperbaric oxygen exposure. Previously, it has been assumed that hyperoxia increases the formation of reactive oxygen species and can reduce the bioavailability of NO, either by rapidly reacting with it (Rubanyi and Vanhoutte 1986) or by impairing the function of NO synthase. Obad et al. (2007a,b) showed that endothelial dysfunction on the brachial artery occurring after diving is partially reversed by acute and long-term predive supplementation of antioxidants, implicating oxidative stress as an important contributor to the postdive endothelial dysfunction. It has also been previously shown that there is a cumulative effect in the level of oxidative stress after successive dives (Obad et al. 2010). In our study, there was no change for mean plasmatic values of NT even after the HBO protocol. These results show nonexistence of oxidative stress mechanisms capable of producing NT. Whether this results from diving acclimatization or physical training in this study remains unknown. It has been demonstrated that regular physical activity increases endogenous antioxidant activity (Elosua et al. 2003) which may explain a better adaptation–compensation response to an increase in reactive oxygen species production (Ji 1995). Another explanation might be that the oxidative stress occurring during the dive did not affect the NO pathway. Assessment of different biomarkers for oxidative stress could be useful to investigate these hypotheses in further experiments.

Laser Doppler measurements after the immersion protocol also recorded no change in microvascular function, excluding the involvement of cold water immersion and moderate physical activity in vascular function alteration that occurs during SCUBA diving. No changes were observed in plasma markers (VWf, PF4, PC, NT) after immersion except that NO level increased significantly. We hypothesize that this increase in NO concentration could be due to the moderate finning exercise performed and which may have been higher than during the SCUBA protocol. The lack of precision in monitoring the exercise intensity level during each protocol can be considered a limitation of this study. A second limitation considered is that the study was performed in limited number of subjects, making it necessary to view the obtained results with caution. However, these results confirm those from a study of recreational divers (Lambrechts et al. 2013).

This study highlighted change in microvascular endothelial-independent but not -dependent function in highly trained divers after a single air dive. The results suggest that the effects of decompression on microvascular endothelium may be modified by diving acclimatization and/or the lower level of bubble formation, both of which may be related. In this regard, postdive alteration of endothelial function may be related to stress of decompression. However, lower bubble formation may produce VSM decompression stress. Further studies are needed to confirm these results both in man and in animal models of DCS.
